# Phosphorylation of FANCD2 Inhibits the FANCD2/FANCI Complex and Suppresses the Fanconi Anemia Pathway in the Absence of DNA Damage

**DOI:** 10.1016/j.celrep.2019.05.003

**Published:** 2019-06-04

**Authors:** David Lopez-Martinez, Marian Kupculak, Di Yang, Yasunaga Yoshikawa, Chih-Chao Liang, Ronghu Wu, Steven P. Gygi, Martin A. Cohn

**Affiliations:** 1 Department of Biochemistry, University of Oxford, Oxford OX1 3QU, UK; 2 Department of Cell Biology, Harvard Medical School, Boston, MA 01125, USA

**Keywords:** Fanconi anemia, interstrand crosslink repair, ICL repair, FANCD2/FANCI, genome stability, DNA repair, CK2, casein kinase 2, phosphorylation, kinase

## Abstract

Interstrand crosslinks (ICLs) of the DNA helix are a deleterious form of DNA damage. ICLs can be repaired by the Fanconi anemia pathway. At the center of the pathway is the FANCD2/FANCI complex, recruitment of which to DNA is a critical step for repair. After recruitment, monoubiquitination of both FANCD2 and FANCI leads to their retention on chromatin, ensuring subsequent repair. However, regulation of recruitment is poorly understood. Here, we report a cluster of phosphosites on FANCD2 whose phosphorylation by CK2 inhibits both FANCD2 recruitment to ICLs and its monoubiquitination *in vitro* and *in vivo*. We have found that phosphorylated FANCD2 possesses reduced DNA binding activity, explaining the previous observations. Thus, we describe a regulatory mechanism operating as a molecular switch, where in the absence of DNA damage, the FANCD2/FANCI complex is prevented from loading onto DNA, effectively suppressing the FA pathway.

## Highlights

• Phosphorylation of FANCD2 suppresses the Fanconi anemia pathway
• FANCD2 is phosphorylated at a cluster between residues 882 and 898
• DNA binding activity of the FANCD2/FANCI complex is reduced upon phosphorylation
• Phosphorylation prevents FANCD2/FANCI recruitment to DNA in the absence of DNA damage

## Graphical Abstract

**Figure:**
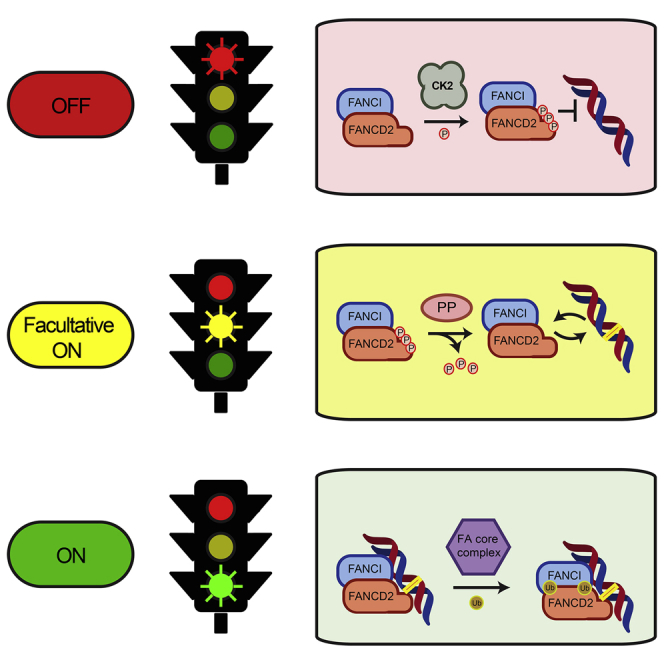

## Teaser

Lopez-Martinez et al. describe a regulatory switch in the Fanconi anemia pathway. Phosphorylation of FANCD2 reduces the DNA binding activity of the FANCD2/FANCI complex, thereby preventing spurious activation of the pathway in the absence of DNA damage.

## Introduction

Our genomes are exposed to multiple sources of DNA damage, both exogenous and endogenous. The cell counts on multiple repair pathways to handle these damages, which are strictly regulated by a complex signaling network involving different post-translational modifications called the DNA damage response.

Interstrand crosslinks (ICLs) are formed when the two strands of DNA are covalently bound after exposure to a crosslinking agent. ICLs can thus block processes that require the opening of the DNA double helix, such as DNA replication and transcription. A complex set of DNA damage response pathways handles ICLs, including the Fanconi anemia (FA)/BRCA (breast cancer genes) repair pathway, translesion synthesis (TLS), homologous recombination (HR), and nucleotide excision repair (NER) (Ceccaldi et al., 2016, Lopez-Martinez et al., 2016) and a separate NEIL3-dependent ICL repair pathway (Semlow et al., 2016).

FA is a rare recessive disorder leading to chromosome instability, developmental abnormalities, bone marrow failure, aplastic anemia, and enhanced susceptibility to certain tumors (Dong et al., 2015, Dufour and Svahn, 2008). Currently, 22 genes have been identified to cause FA, and they all code for proteins implicated in the repair of ICLs. Mutations in several of these genes have also been linked to increased incidence of certain cancers, for example, BRCA1, BRCA2 and PALB2 (Deans and West, 2011, King et al., 2003).

The repair of the ICL can be divided in three main parts: the detection of the damage by sensors and the early activation of the pathway through recruitment of the FA core complex, followed by the recruitment and activation of FANCD2/FANCI, which in turn coordinates the activity of downstream effectors that perform the repair. The first of the sensors found was the complex formed by FANCM, FAAP24, and MHF1/2, which is recruited to replication forks stalled by ICLs (Ciccia et al., 2007, Huang et al., 2013, Yan et al., 2010, Zhao et al., 2014). This complex was then proposed to promote the recruitment of the FA core complex, which contains 14 proteins: FANCA, FANCB, FANCC, FANCE, FANCF, FANCG, FANCL, FANCM, FANCT, FAAP100, MHF1, MHF2, FAAP20, and FAAP24. Recently, two proteins, UHRF1 and UHRF2, have been found to also function as ICL sensors, which are recruited to the lesion within seconds and promote activation of the pathway (Liang et al., 2015, Motnenko et al., 2018). Stalling of two opposing replication forks at the ICL is also believed to contribute to activation of repair (Zhang et al., 2015). After detection of the ICL, an essential step of the pathway is the recruitment of the FANCD2/FANCI complex. The FANCD2/FANCI complex is recruited to the lesion through its DNA binding capacity, but this is also promoted through its interactions with UHRF1, UHRF2, and some components of the FA core complex, mainly the E3 ligase FANCL, which together with the E2 conjugating enzyme FANCT or UBE2T monoubiquitinates FANCD2 and FANCI (Hodson et al., 2014, Machida et al., 2006, Meetei et al., 2003). FANCD2/FANCI is then thought to regulate the recruitment of the nuclease complex, XPF/ERCC1, which makes incisions around the ICL leading to its unhooking (Klein Douwel et al., 2014, Kuraoka et al., 2000, Yamamoto et al., 2011). The repair is finally completed by a combination of TLS, HR, and NER.

Monoubiquitination of FANCD2/FANCI is necessary for the progress of the pathway and subsequent repair (Sims et al., 2007, Smogorzewska et al., 2007). Because mutant forms of FANCD2/FANCI unable to be monoubiquitinated completely abrogated recruitment, it seemed that monoubiquitination should precede DNA binding. However, *in vitro* studies showed that the monoubiquitination reaction could be greatly enhanced by the presence of DNA, pointing to the reaction’s taking place on the DNA (Longerich et al., 2014, Rajendra et al., 2014, Sato et al., 2012). Recent studies have also identified several positively charged residues in the C-terminal domain of FANCD2, the Tower domain, that are necessary for DNA binding and for the monoubiquitination to take place in cells (Liang et al., 2016). Therefore, DNA binding most likely precedes monoubiquitination. Simultaneously, monoubiquitination is crucial for the retention of FANCD2/FANCI on chromatin. However, these protein-DNA interactions are static and do not provide for a regulated recruitment in response to DNA damage. Thus, the question remains how DNA binding by FANCD2/FANCI is regulated.

For efficient and controlled repair to take place, which avoids spurious events, strict regulatory steps must be in place. The first master regulator of the FA pathway was found to be the ATR kinase (ATM and Rad3-related) (Andreassen et al., 2004, Pichierri and Rosselli, 2004). ATR phosphorylates several components of the core complex, such as FANCA, leading to promotion of FANCD2 monoubiquitination and ICL repair (Collins et al., 2009). ATR can also directly phosphorylate FANCD2 and FANCI, promoting their monoubiquitination, leading to an activation of the FA pathway, though the precise mechanism behind these activating phosphorylation events remains unclear (Andreassen et al., 2004, Cheung et al., 2017, Ishiai et al., 2008, Taniguchi et al., 2002, Zhi et al., 2009).

Here we report a previously unknown phosphorylation event on FANCD2 at a six-residue cluster (S882, T884, S886, S891, T896, and S898) catalyzed by the kinase CK2 (casein kinase 2). We found that phosphorylation of these sites on FANCD2 led to increased sensitivity to crosslinking agents, inhibited monoubiquitination, and abrogated recruitment to ICLs in human cells. This phosphorylation event also led to inhibition of monoubiquitination *in vitro*. Furthermore, we describe a mechanism whereby the phosphorylation of FANCD2 at these sites reduces FANCD2/FANCI DNA binding activity, blocking subsequent steps in the pathway. Lack of phosphorylation on these sites leads to enhanced recruitment of FANCD2/FANCI to ICLs and increased monoubiquitination. This mechanism could thus act as a safeguard to avoid any undesired or exacerbated recruitment of FANCD2/FANCI complex to the genome.

## Results

### FANCD2 Is Phosphorylated on Residues S882, T884, S886, S891, T896, and S898 *In Vivo*

**Figure 1:**
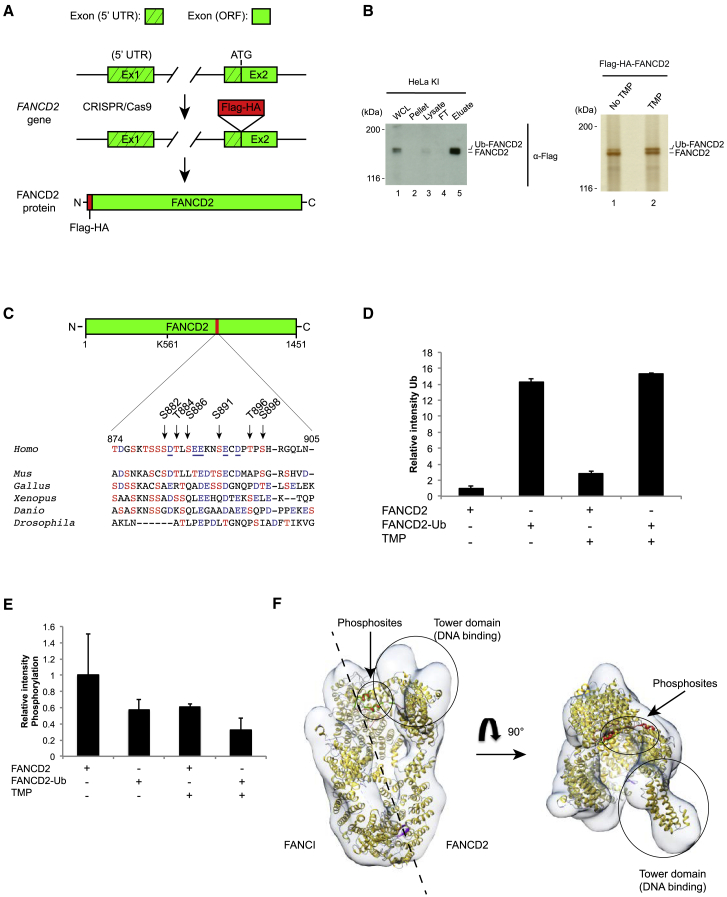
Identification of a Phosphorylation Cluster on FANCD2 (A) Schematic representation of the generation of a Flag-HA-FANCD2 knock-in HeLa cell line through the use of CRISPR/Cas9. Exons are shown in green, and Flag-HA tag is shown in red. (B) Flag purification of both FANCD2 and Ub-FANCD2 from the knock-in HeLa cell line. Immunoblot analysis showing whole-cell lysate (WCL), pellet, lysate, flowthrough (FT), and elution of the purification. Silver stain showing the four elution products used for MS/MS in unperturbed conditions (no TMP) and after the induction of ICLs with TMP (TMP). (C) Alignment of residues 874–905 of human FANCD2 protein to those in mouse (*Mus*), chicken (*Gallus*), toad (*Xenopus*), zebrafish (*Danio*), and fruit fly (*Drosophila*). Serine and threonine residues are in red, and aspartic and glutamic acid residues are in blue (alignments done with ClustalW2). (D) Relative intensity of ubiquitinated peptides to unmodified peptides as identified in MS/MS in four samples: no ubiquitination/no TMP (1), ubiquitination/no TMP (2), no ubiquitination/TMP (3), and ubiquitination/TMP (4). Mean ± SD in n = 2 independent experiments. (E) Relative intensity of phosphorylated peptides to unmodified peptides (containing residues 882, 884, 886, 891, 896, and 898) as identified in MS/MS in four samples: no ubiquitination/no TMP (1), ubiquitination/no TMP (2), no ubiquitination/TMP (3), and ubiquitination/TMP (4). Mean ± SD in n = 2 independent experiments. (F) Crystal structure of the mouse FANCD2/FANCI complex (marked in yellow; PDB: 3S4W) docked into the cryo-EM structure of the human FANCD2/FANCI complex (in gray; EMDB: EMD-8141) showing the approximate location of the 882–898 cluster (marked in red) as well as the C-terminal Tower domain of FANCD2, where some DNA binding residues have been found. It should be noted that residues 882–898 were deleted from the mouse FANCD2 protein used to obtain the crystal structure.

In order to study phosphorylation events in FANCD2, we introduced a Flag-HA tag into the *FANCD2* gene in HeLa cells using CRISPR/Cas9, creating an N-terminal-tagged fusion protein (Figures 1A and S1A). We then introduced ICLs in the cells and purified Flag-HA-FANCD2 (Figure 1B). Induction of monoubiquitinated FANCD2 was robust compared with untreated control cells. Tandem mass spectrometry (MS/MS) analysis of the purified protein revealed phosphorylation of multiple residues, including residues previously reported—S1257, S1401, S1404, and S1407 (Taniguchi et al., 2002)—underscoring the validity of the experiment (Figure S1B). We also discovered the existence of a new phosphorylation cluster, spanning amino acids 882–898. We identified six phosphorylated amino acids in the cluster, several of which are well conserved (Figures 1C, S1B, and S1C). Some acidic residues in the cluster are also well conserved. On the basis of the amino acid sequence, the predicted kinase responsible for phosphorylation of this cluster is CK2 (Pinna, 2002) (GPS 2.1 and NetPhos 3.1). Mass spectrometry can sometimes provide insight into dynamics of post-translational modifications, although quantitative determination is not always possible (Presler et al., 2017). To test whether our data could give such information, we first assessed the degree of monoubiquitination in the four samples analyzed (FANCD2 and Ub-FANCD2, either before or after the introduction of ICLs). We cut out and extracted each of these four bands of a gel similar to the one shown in Figure 1B, but now containing more protein, and stained with Coomassie blue. The level of ubiquitination in the four samples was assessed as a control for the potential contamination between samples because of incomplete separation of the bands in SDS-PAGE. Indeed, a clear enrichment in monoubiquitination could be observed in the samples containing Ub-FANCD2 compared with the other samples, suggesting that the separation was sufficient (Figure 1D). We then assessed whether a change in phosphorylation of the 882–898 cluster could be observed. A decrease in phosphorylation of the cluster was observed in the monoubiquitinated FANCD2 protein after the introduction of ICLs (Figure 1E). Examination of the crystal structure of the mouse FANCD2/FANCI complex (Joo et al., 2011) and the cryoelectron microscopy (cryo-EM) structure of the human FANCD2/FANCI complex (Liang et al., 2016) suggests that the 882–898 phosphorylation cluster is located on the surface of the inner cavity of the heterodimer, potentially in contact with DNA (Figure 1F). Taken together, our data suggest that phosphorylation of the 882–898 cluster is associated with the complex in its non-ubiquitinated form, while the dephosphorylated state is associated with the complex in its ubiquitinated form.

### Phosphorylation of the FANCD2 882–898 Cluster Reduces the Activity of the FANCD2/FANCI Complex *In Vivo*

**Figure 2:**
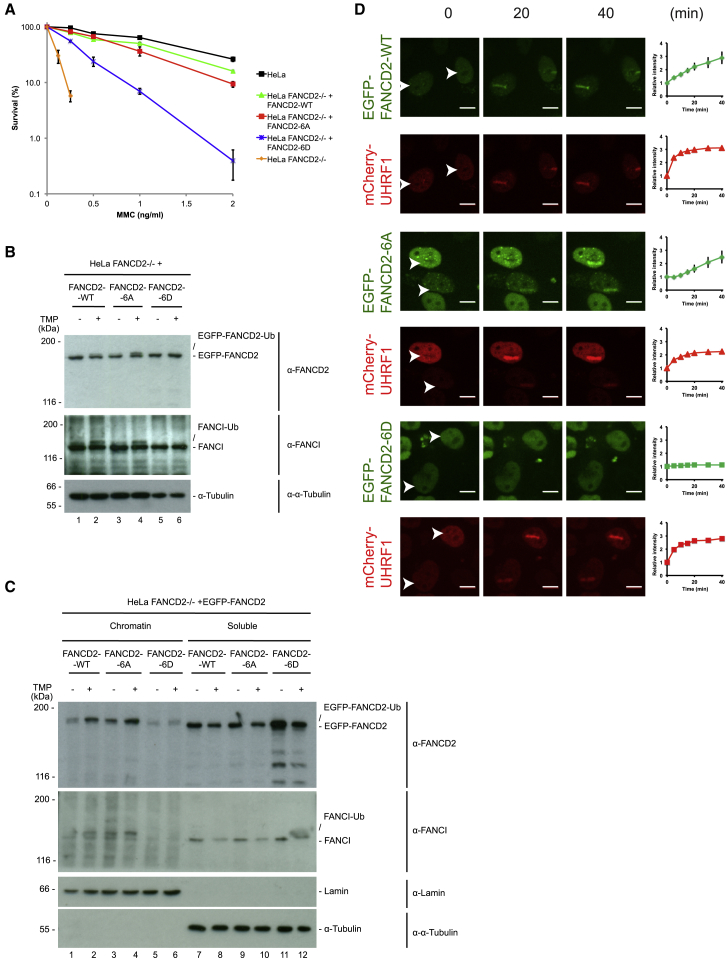
Phosphorylation of the 882–898 Cluster on FANCD2 Suppresses Activation of the FANCD2/FANCI Complex in Human Cells (A) Survival assay to the crosslinking agent mitomycin C (MMC) added at the indicated concentrations and left for 2 weeks. Survival is assessed as the number of colonies formed after 2 weeks. HeLa cells were used, FANCD2 was knocked out with CRISPR/Cas9, and the HeLa FANCD2−/− cells were stably complemented with EGFP-FANCD2, EGFP-FANCD2-6A, or EGFP-FANCD2-6D (mean ± SEM, n = 3). (B) Immunoblot analysis of cell lysates of the HeLa FANCD2−/− cells complemented with EGFP-FANCD2, EGFP-FANCD2-6A, or EGFP-FANCD2-6D before and after treatment with TMP (2 μg/mL) and UVA (50 mJ/cm^2^) for 3 h. (C) Immunoblot analysis of cell lysates of HeLa FANCD2−/− cells complemented with EGFP-FANCD2, EGFP-FANCD2-6A, or EGFP-FANCD2-6D before and after treatment with TMP (2 μg/mL) and UVA (50 mJ/cm^2^) for 3 h. Samples were fractionated with CSK buffer so chromatin-bound and soluble fractions could be separated. (D) Live cell imaging of HeLa FANCD2−/− cells complemented with EGFP-FANCD2, EGFP-FANCD2-6A, or EGFP-FANCD2-6D and mCherry-UHRF1. Cells were treated with TMP (20 μg/mL) and microirradiated at the indicated areas (white arrows) and followed for the indicated times (stripe intensity quantified as mean ± SEM, n = 5) (scale bar, 10 μm).

We decided to test whether the 882–898 phosphorylation cluster is functionally important for the activity and regulation of the FANCD2/FANCI complex. To this end, we depleted endogenous FANCD2 from HeLa cells using CRISPR/Cas9, generating HeLa FANCD2−/− cells. This cell line was then complemented with either EGFP-FANCD2, or mutant derivatives in which the six phosphorylation sites in the cluster have been mutated to either alanines to prevent phosphorylation, or to aspartic acid residues to mimic constitutive phosphorylation, EGFP-FANCD2-6A and EGFP-FANCD2-6D, respectively. Importantly, all three proteins were expressed at the same levels as endogenous FANCD2 (Figure S2A). The resulting cell lines were then subjected to a clonogenic survival assay under increasing concentrations of the ICL-inducing drug mitomycin C (MMC). As expected, HeLa FANCD2−/− cells were extremely sensitive (Figure 2A). EGFP-FANCD2 restored resistance to MMC, whereas EGFP-FANCD2-6A displayed very mild sensitivity. On the other hand, EGFP-FANCD2-6D was significantly less functional, suggesting that phosphorylation of the cluster leads to reduced activity of the FANCD2/FANCI complex.

To gain insight into the underlying mechanism, we assessed the abilities of the two versions of FANCD2 to be monoubiquitinated *in vivo*. Again, EGFP-FANCD2 and EGFP-FANCD2-6A were monoubiquitinated normally and complemented monoubiquitination of endogenous FANCI (Figure 2B). On the contrary, EGFP-FANCD2-6D was not monoubiquitinated and did not complement monoubiquitination of FANCI. We also noticed that EGFP-FANCD2-6A was monoubiquitinated slightly stronger than EGFP-FANCD2 (Figure 2B, lanes 4 and 2, respectively). We speculated that EGFP-FANCD2-6D was not recruited properly to chromatin, a defect that could lead to the observed phenotype of defect in monoubiquitination. We therefore biochemically fractionated cells after the induction of ICLs and assessed the localization of the non-modified and the monoubiquitinated forms of FANCD2. Although EGFP-FANCD2 and EGFP-FANCD2-6A were correctly localized in the chromatin fraction (Figure 2C, lanes 1–4), EGFP-FANCD2-6D failed to localize properly (Figure 2C, lanes 5 and 6). Similarly, endogenous FANCI also failed to localize to chromatin in the EGFP-FANCD2-6D-expressing cells and showed no signs of monoubiquitination (Figure 2C). To further confirm these observations, we decided to assess the recruitment of the proteins to ICLs in real time in live cells. mCherry-UHRF1 was co-expressed in the three HeLa cell lines described as a control for the introduction of ICLs (Figure S2A). In the absence of TMP, microirradiation alone did not trigger the recruitment of either EGFP-FANCD2 or mCherry-UHRF1 (Figure S2B). ICLs were then introduced by TMP treatment followed by microirradiation, and the recruitment of FANCD2 and UHRF1 was monitored over time. Both EGFP-FANCD2 and EGFP-FANCD2-6A were recruited to ICLs, while EGFP-FANCD2-6D was completely defective in recruitment (Figure 2D). mCherry-UHRF1 was recruited equally in all three cell lines.

Held together, the phosphomimetic FANCD2 protein is not fully functional in complementation assays, is not monoubiquitinated, and is not recruited to ICLs in chromatin.

### CK2 Phosphorylates FANCD2 on the 882–898 Cluster *In Vivo* and Prevents Monoubiquitination *In Vitro*

**Figure 3:**
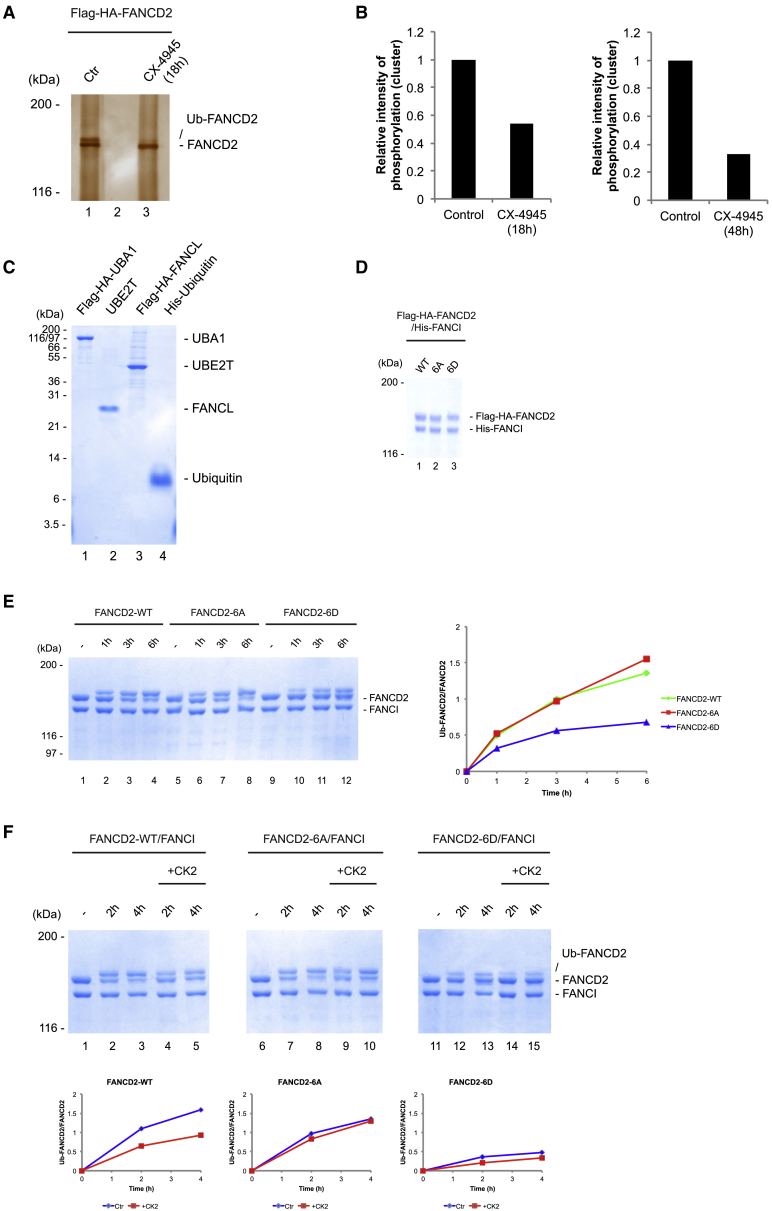
CK2 Phosphorylates FANCD2 in the 882–898 Cluster *In Vivo* and Reduces Its Monoubiquitination *In Vitro* (A) Flag purification of endogenous FANCD2 from HeLa S3 knock-in cell line. Ctr, untreated control; CX-4945, treated with 10 μM CK2 inhibitor CX-4945 for 18 h. (B) Relative intensity of phosphorylated peptides containing the 882–898 cluster on FANCD2 in either untreated control or treated with the CK2 inhibitor CX-4945 for 18 or 48 h. (C) Coomassie blue gel of the proteins used in the *in vitro* ubiquitination assay: Flag-HA-UBA1 (E1), UBE2T (E2), Flag-HA-FANCL (E3), and His-ubiquitin. (D) Coomassie blue gel of the purification of the Flag-HA-FANCD2/His-FANCI complex co-expressed in Sf9 cells. (E) Coomassie blue gel of the *in vitro* ubiquitination of the FANCD2/FANCI complex as WT, 6A, or 6D forms. Quantification showing the ratio of Ub-FANCD2 to FANCD2. (F) Coomassie blue gel of the *in vitro* ubiquitination of the WT, 6A, and 6D forms of the FANCD2/FANCI complex following a mock treatment or *in vitro* phosphorylation by CK2. Quantification showing the ratio of Ub-FANCD2 to FANCD2.

As previously mentioned, the sites on the 882–898 cluster on FANCD2 are predicted to be phosphorylated by CK2. In order to test this hypothesis *in vivo*, we treated HeLa S3 knock-in cells expressing endogenous Flag-HA-FANCD2 with a potent and specific CK2 inhibitor, CX-4945 (Son et al., 2013), purified Flag-HA-FANCD2, and analyzed the purified protein using MS/MS (Figure 3A; Figure S3A). We could observe a reduction in the relative intensity of phosphorylation of the cluster to about 50% after 18 h treatment with the inhibitor (Figure 3B), while other phosphorylation sites on FANCD2 remained unaffected (Figure S3B). After 48 h inhibition of CK2, a further reduction to about 30% in phosphorylation of the cluster could be observed, but this long treatment seemed to indirectly affect other phosphosites on FANCD2 as well, though to a lesser extent (Figure 3B; Figure S3B).

Our results suggest that dynamic phosphorylation and dephosphorylation are controlling the activation of the FANCD2/FANCI complex. To gain deeper mechanistic insight into this regulatory switch, we turned to *in vitro* experimentation. More specifically, we decided to fully reconstitute the monoubiquitination reaction *in vitro*, allowing us to dissect and uncover exactly where in the reaction phosphorylation of the FANCD2 cluster plays a role. We fully reconstituted the monoubiquitination reaction *in vitro*, using purified components (Figure 3C). We then purified the FANCD2/FANCI, FANCD2-6A/FANCI, and FANCD2-6D/FANCI complexes to homogeneity in order to assess their activities as substrates in the reaction (Figure 3D). As expected, the FANCD2/FANCI complex was efficiently monoubiquitinated in the reaction (Figure 3E, lanes 1–4). Likewise, the FANCD2-6A/FANCI complex was also monoubiquitinated with similar kinetics (Figure 3E, lanes 5–8). In contrast, and in good agreement with the *in vivo* data, the FANCD2-6D/FANCI complex was only weakly monoubiquitinated and with slower kinetics (Figure 3E, lanes 9–12).

These data suggest that the FANCD2/FANCI complex is kept in an inactive state through constitutive phosphorylation of the 882–898 cluster by CK2. To test this hypothesis directly, we assessed whether CK2 can reduce the activity of the FANCD2/FANCI complex in our *in vitro* monoubiquitination assay. We phosphorylated the FANCD2/FANCI complex using recombinant CK2 and then subjected the complex to the *in vitro* monoubiquitination assay. We observed a clear reduction in monoubiquitination after CK2 treatment (Figure 3F, lanes 4 and 5). As expected, the FANCD2-6A/FANCI complex was unaffected by the CK2 treatment (Figure 3F, lanes 9 and 10), underscoring the specificity.

Taken together, these data show that the FANCD2/FANCI complex is kept in an inactive state through constitutive phosphorylation of the 882–898 cluster by CK2.

### Phosphorylation of the 882–898 Cluster in FANCD2 Abrogates DNA Binding of the FANCD2/FANCI Complex

The inhibitory effect that phosphorylation of the 882–898 cluster in FANCD2 has on monoubiquitination of the FANCD2/FANCI complex could be caused by at least three different mechanisms. First, the integrity of the FANCD2/FANCI complex could be affected, causing dissociation. This could be causative, because monoubiquitination of FANCD2 is dependent on its heterodimerization with FANCI (Liang et al., 2016, van Twest et al., 2017). Second, the ability of the complex to interact with the FANCL E3 ligase could be affected. Third, the ability of the complex to interact with DNA could be affected. The latter would be causative, because interaction of the complex with DNA is required for proper monoubiquitination (Liang et al., 2016). We decided to test if any of these three options constituted the mechanism of inhibition.

**Figure 4:**
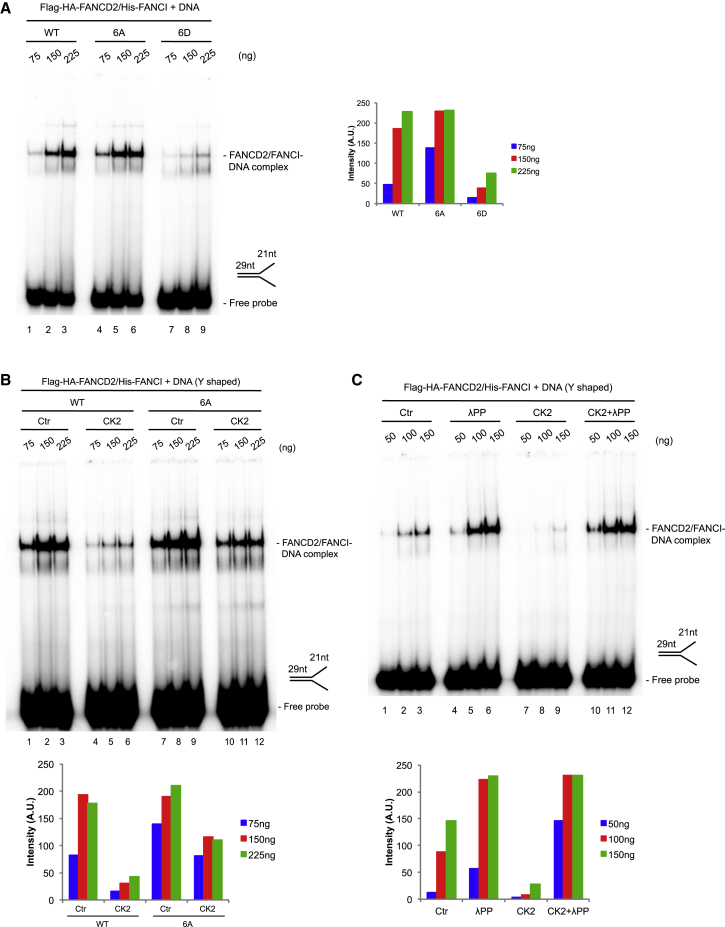
Phosphorylation of the 882–898 Cluster on FANCD2 Hinders Its DNA Binding and Can Be Reversed by Dephosphorylation (A) Electrophoretic mobility shift assay (EMSA) showing the DNA binding of the Flag-HA-FANCD2/His-FANCI complex (WT, 6A, or 6D form) to a Y-shaped radiolabeled DNA probe (representative experiment of n = 2). Quantification showing intensities of the protein/DNA complexes. (B) EMSA showing the DNA binding of the Flag-HA-FANCD2/His-FANCI complex (WT and 6A) after mock or CK2 treatment to a Y-shaped radiolabeled DNA probe (loading control in Figure S4A) (representative experiment of n = 2). Quantification showing intensities of the protein/DNA complexes. (C) EMSA showing the DNA binding of the Flag-HA-FANCD2/His-FANCI complex (WT) after mock, CK2, λPP or both treatments to a Y-shaped radiolabeled DNA probe (representative experiment of n = 2). Quantification showing intensities of the protein/DNA complexes.

First, the FANCD2-6A/FANCI and FANCD2-6D/FANCI complexes were as stable as the FANCD2/FANCI complex, showing that integrity of the complexes was not the mechanism (Figure 3D). Second, we tested the interaction of the three complexes with recombinant FANCL. We found an indistinguishable interaction of the complexes with FANCL (Figure S4A). Third, we assessed the ability of the three complexes to bind DNA. Here we applied an electrophoretic mobility shift assay, using a radiolabeled DNA structure mimicking a stalled replication fork. The FANCD2/FANCI complex formed a strong protein/DNA complex (Figure 4A, lanes 1–3). Similarly, the FANCD2-6A/FANCI complex possessed similar DNA binding properties (Figure 4A, lanes 4–6). In contrast, the FANCD2-6D/FANCI complex was almost defective in DNA binding (Figure 4A, lanes 7–9).

We then tested whether phosphorylation of FANCD2 by CK2 could also reduce the DNA binding activity of the FANCD2/FANCI complex. Phosphorylated FANCD2 showed a marked reduction in DNA binding compared with the mock treated control (Figure 4B, lanes 1–3 and 4–6). To assess the specific role of the 882–898 cluster in this effect, we also compared CK2 phosphorylated and mock treated FANCD2-6A/FANCI. We observed a less pronounced defect in DNA binding of the phosphorylated FANCD2-6A/FANCI compared with the phosphorylated FANCD2-WT (wild-type)/FANCI (Figure 4B, lanes 4–6 and 10–12). However, the CK2 phosphorylation of the FANCD2-6A/FANCI still reduced the affinity for DNA compared with the mock treatment (Figure 4B, lanes 7–9 and 10–12). None of these effects were due to differences in protein amounts (Figure S4B). These results point to the 882–898 cluster’s playing an important role in the DNA binding activity of the complex, but they also show that, at least *in vitro*, other sites are likely phosphorylated by CK2. It remains unknown whether these putative additional phosphosites are relevant *in vivo*.

Finally, we decided to test whether this phosphorylation event could be reversed through dephosphorylation and thus recapitulate *in vitro* the potential *in vivo* switch mechanism. After phosphorylating FANCD2/FANCI with CK2, we dephosphorylated the resulting product with the broad-specificity lambda protein phosphatase (λPP). We could observe a clear reduction in DNA binding after CK2 treatment (Figure 4C, lanes 1–3 [mock treated] and 7–9 [CK2 treated]), which could be reversed by λPP treatment (Figure 4C, lanes 7–9 [CK2 treated] and 10–12 [CK2 followed by λPP treatment]). In fact, DNA binding after λPP treatment was increased compared with the mock treated control (Figure 4C, lanes 1–3 [mock treated] and 4–6 [λPP treated]), pointing to partial phosphorylation already present in the FANCD2/FANCI complex purified from Sf9 cells. This result shows that the negative effect on DNA binding by CK2 phosphorylation can be reversed through dephosphorylation *in vitro*, pointing to a dynamic regulatory process *in vivo*.

Taken together, these data demonstrate that phosphorylation of the 882–898 cluster in FANCD2 by CK2 reduces binding of the FANCD2/FANCI complex to DNA, in turn preventing its activation by monoubiquitination. Furthermore, dephosphorylation of the phosphorylated FANCD2/FANCI complex can rescue the DNA binding capacity of the complex, allowing its activation by monoubiquitination.

### Lack of Phosphorylation of the 882–898 Cluster on FANCD2 Does Not Affect the Cell Cycle Distribution but Leads to an Exacerbated Response to ICLs

**Figure 5:**
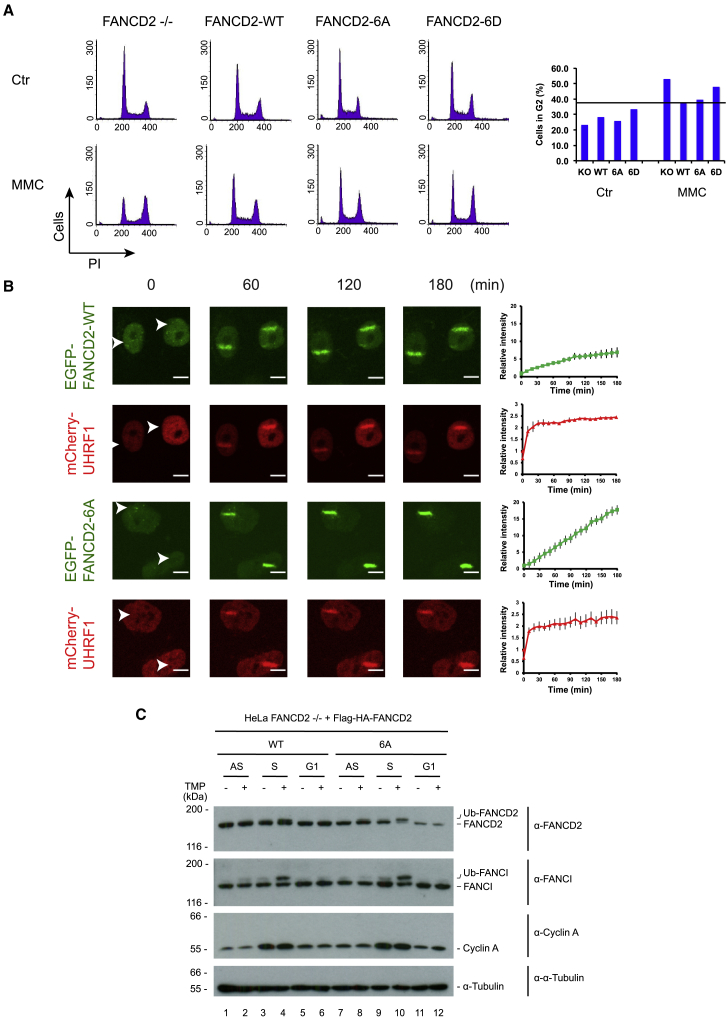
Lack of Phosphorylation of the 882–898 Cluster on FANCD2 Leads to Enhanced Recruitment to ICLs and Ubiquitination *In Vivo* (A) Cell cycle profiles measured by DNA content of HeLa FANCD2−/− cells and HeLa FANCD2−/− cells complemented with FANCD2-WT, FANCD2-6A, and FANCD2-6D after no treatment (Ctr) or after treating with 20 ng/mL MMC for 2 h and recovering for 24 h (MMC). Graph shows the percentage of cells in G2 for each cell line and treatment. (B) Live-cell imaging of HeLa FANCD2−/− cells complemented with EGFP-FANCD2 or EGFP-FANCD2-6A and mCherry-UHRF1. Cells were treated with TMP (20 μg/mL) and microirradiated at the indicated areas (white arrows) and followed for the indicated times (stripe intensity quantified as mean ± SEM, n = 5) (scale bar, 10 μm). (C) Immunoblot analysis of cell lysates of HeLa FANCD2−/− cells complemented with Flag-HA-FANCD2 or Flag-HA-FANCD2-6A either asynchronous (AS) or synchronized with double thymidine block (S and G1). ICLs were introduced with TMP (2 μg/mL) and UVA (50 mJ/cm^2^) 1.5 h before harvest.

Because CK2 phosphorylation of FANCD2 regulates the DNA binding and subsequent activation of the FANCD2/FANCI complex, it is reasonable to speculate that lack of phosphorylation on the 882–898 cluster of FANCD2 could lead to an exacerbated, uncontrolled response of the complex. First, we decided to test the effect of the phosphorylation status of FANCD2 in the cell cycle progression of both unperturbed cells and MMC-treated cells. It has been long known that FA cells suffer from increased G2 arrest after treatment with MMC, and this is actually a defining phenotype of FA cells (Kaiser et al., 1982). Whereas in unperturbed conditions only small changes in the percentage of cells in G2 could be observed, after MMC exposure, HeLa FANCD2−/− cells had an additional 15% G2 arrest compared with FANCD2-WT complemented cells. FANCD2-6D complemented cells displayed an additional 10% G2 arrest (Figure 5A). However, FANCD2-6A complemented cells had a similar rescued phenotype to FANCD2-WT complemented cells (Figure 5A), overall a similar result to the survival assay to MMC (Figure 2A).

If our interpretation of the data is correct, then the non-phosphorylatable derivative of FANCD2, FANCD2-6A, should not be suppressed by CK2 in live cells. If so, we would predict that recruitment of this protein to ICLs should be uncontrolled, because it no longer can be suppressed. We decided to test this directly. We introduced ICLs in cells and followed the live recruitment of EGFP-FANCD2 and EGFP-FANCD2-6A over a longer time course (3 h). We could observe an accumulation of EGFP-FANCD2-WT to 6-fold over the nucleus intensity, plateauing toward the 2 h time point (Figure 5B). In contrast, EGFP-FANCD2-6A continued to accumulate to 17-fold 3 h after the introduction of ICL, with no signs of reduction in recruitment during the time course. UHRF1 was recruited to a similar extent in both cases (Figure 5B).

We also assessed the recruitment kinetics of EGFP-FANCD2 and EGFP-FANCD2-6A in a more traditional way, by monitoring nuclear foci. In order to accurately determine the cell cycle stage of each cell, we stably expressed the mCherry-geminin-1-110 fusion protein in both cell lines. This fusion protein contains the first 110 amino acids of geminin, which contains a degron domain, fused to mCherry. Therefore, the fusion protein is present only in S-phase cells (Sakaue-Sawano et al., 2008). Visualization and determination of S-phase cells (mCherry-geminin-1-110 fusion-positive cells) was clear (Figure S5A). Induction of ICLs with TMP + UVA treatment led to a marked increase in the number of FANCD2-WT and FANCD2-6A foci both 3 and 16 h after damage, with slightly more FANCD2-6A foci compared with FANCD2-WT (Figure S5B). Also, the total area of FANCD2-6A foci was increased by 60 units 3 h after ICL induction compared with an increase of 30 units for FANCD2-WT. Additionally, the total area of FANCD2-6A foci at 3 h was 120 units compared with 90 units for FANCD2-WT (Figure S5C). Taken together, this overall increase in number and area of FANCD2-6A foci recapitulates the observed uncontrolled recruitment to ICLs induced by microirradiation (Figure 5B).

Increased FANCD2 recruitment to ICLs in S-phase cells could result in enhanced monoubiquitination of FANCD2. We decided to check this directly in synchronized HeLa cells. We synchronized HeLa FANCD2−/− cells complemented with either Flag-HA-FANCD2 or Flag-HA-FANCD2-6A using a double thymidine block (Figure S5D) and assessed monoubiquitination of FANCD2 and FANCI 1.5 h after introduction of ICLs. As expected, FANCD2-WT was mildly monoubiquitinated in S-phase cells but not visibly monoubiquitinated in asynchronous and G1-phase cells (Figure 5C, lanes 2, 4, and 6). In contrast, FANCD2-6A, was visibly monoubiquitinated in both asynchronous and S-phase cells but also not in G1-phase cells (Figure 5C, lanes 8, 10, and 12). The degree of monoubiquitination of FANCD2-6A compared with FANCD2 was particularly striking in S-phase cells. Here, the majority of FANCD2-6A was monoubiquitinated compared with a minority in FANCD2 (Figure 5C, compare lanes 4 and 10). This further supports the previous observations that expressing FANCD2-6A led to increased recruitment and foci extension after ICL induction.

In conclusion, these data suggest that a non-phosphorylated 882–898 cluster creates a facultative active form of the FANCD2/FANCI complex, which therefore responds more quickly and strongly to DNA damage than the phosphorylated counterpart. Phosphorylation is necessary to control appropriate recruitment and activation of the FANCD2/FANCI complex.

## Discussion

**Figure 6:**
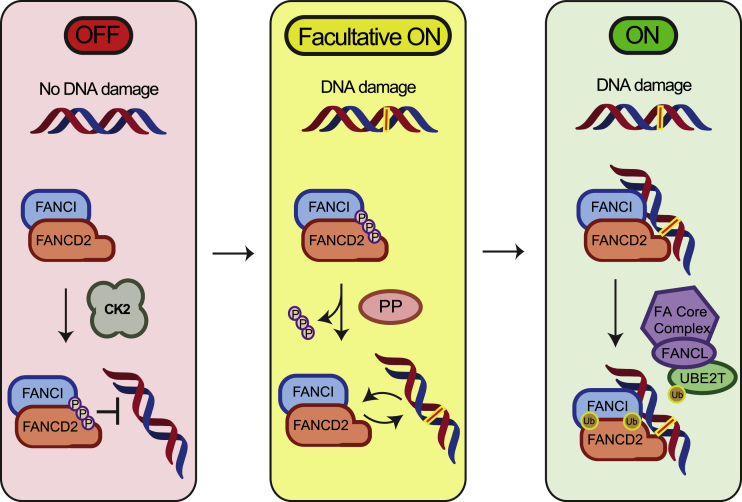
Model of the Regulation of the FA Pathway by CK2 Phosphorylation of the FANCD2/FANCI Complex CK2 phosphorylates FANCD2 constitutively on the 882–898 cluster and prevents DNA binding of the FANCD2/FANCI complex in the absence of DNA damage. Upon the appearance of DNA damage, FANCD2 is dephosphorylated, increasing the affinity of the FANCD2/FANCI complex to DNA. This form of the FANCD2/FANCI complex can be considered facultative active. Once bound to DNA, the FANCD2/FANCI complex is monoubiquitinated by the core complex containing the E3 ligase FANCL, bringing the complex to its fully active state. Monoubiquitination locks the FANCD2/FANCI complex on DNA, completing its activation, and allows ICL repair to initiate.

We have shown that phosphorylation of the 882–898 cluster on FANCD2 greatly reduces the affinity of the FANCD2/FANCI complex for DNA, thereby keeping the complex away from DNA in the absence of genotoxic stress. We propose that CK2 is the main kinase responsible for this phosphorylation event, although other kinases could also contribute, perhaps depending on the stage of the cell cycle or the stimulus triggering the phosphorylation. This model entails that the regulated event upon DNA damage is dephosphorylation of FANCD2/FANCI by a still unidentified phosphatase that creates a facultative active form of the FANCD2/FANCI complex with high affinity for DNA. This form of the complex is not yet fully active but can now be loaded onto DNA, where further modifications and full activation can take place, mainly via monoubiquitination by FANCL but possibly also positively acting phosphorylation events by ATR (Figure 6). The outcome of these modifications, potentially also entailing a conformational change of the complex, is its stable locking on DNA and its subsequent engagement in repair. In this way the binding of FANCD2/FANCI to DNA could be regulated prior to its monoubiquitination and act as a safeguard against spurious activation of DNA repair pathways involving the FANCD2/FANCI complex.

This phosphorylation event could also play a role in the shutdown of the pathway and the eviction of the FANCD2/FANCI complex from DNA. However, we still do not know exactly when (re)phosphorylation takes place. It could happen while the complex is still bound to DNA and promote its dissociation from DNA, either before or after deubiquitination by the USP1/UAF1 deubiquitinating enzyme complex and thereby limit (re)association and reactivation during shutdown of repair.

### Role of Dephosphorylation in the DNA Damage Response

Dephosphorylation has traditionally been associated with shutdown of the DNA damage response once the repair is complete because the master regulators of the DNA damage response are kinases such as ATR and ATM, which are linked to the activation of repair factors in the initial stages of DNA repair (Blackford and Jackson, 2017). However, dephosphorylation has also been shown to be key in the activation of certain pathways, and some phosphatases have already been identified as promoting the DNA damage response (Zheng et al., 2015). The phosphatase PP4C, for example, dephosphorylates 53BP1 to allow its recruitment onto double-strand breaks (DSB) by promoting 53BP1 interaction to ubiquitinated H2A (Lee et al., 2014). In contrast, it was later found that PP4C could also promote HR by dephosphorylating 53BP1 and releasing RIF1, a process dependent on BRCA1 (Isono et al., 2017). In yeast, the phosphatase Cdc14 dephosphorylates the nuclease Yen1, promoting its nuclear localization and increasing its DNA binding affinity in mitosis, where it acts to resolve Holliday junctions (Arter et al., 2018, Blanco et al., 2014).

### Role of CK2 in the DNA Damage Response

Inactivation of the FANCD2/FANCI complex by constitutive phosphorylation by CK2 is a new regulatory step in the FA pathway, but CK2 has already been linked to other DNA repair pathways. Phosphorylation of XRCC1 by CK2 was implicated in single-strand break (SSB) repair and was later shown to promote chromatin dissociation allowing XRCC1 to form protein repair complexes and allowing the incision step necessary for BER (Dutta et al., 2017, Loizou et al., 2004, Ström et al., 2011).

Phosphorylation of H2AX is one of the first events following the generation of a DSB (Rogakou et al., 1998). CK2 was found to mediate an even earlier event, the phosphorylation of HP1β (heterochromatin protein 1β) (Ayoub et al., 2008). Phosphorylation of HP1β on T53 by CK2 led to its release from H3K9me, which in turn promotes chromatin relaxation and downstream events such as γH2AX phosphorylation (Ayoub et al., 2008). For such a quick regulatory event to happen, CK2 must be recruited very early to DSBs, and some evidence for this was later found (Olsen et al., 2012), though the exact mechanism or the mediators remain unknown. Also during DSB repair, CK2 has been shown to constitutively phosphorylate MDC1 promoting the recruitment of the MRN (MRE11-RAD50-NBS1) complex to the sites of damage through its interaction with NBS1 (Chapman and Jackson, 2008, Melander et al., 2008, Spycher et al., 2008). CK2 is also required for the accumulation of 53BP1 at sites of damage (Guerra et al., 2014), and recently CK2 was reported to play an important role for the functional interaction between MDC1 and TOPBP1 during mitosis (Leimbacher et al., 2019). It is through the phosphorylation of LSD1 by CK2, reversible by WIP1, that the ubiquitination of 53BP1 by RNF168 is promoted and 53BP1 recruitment enhanced (Peng et al., 2015). CK2 was also found to be part of the sequential phosphorylation of Rad51, following phosphorylation by Plk1, which leads to the recruitment of Rad51 to DSB through its interaction with NBS1 in a BRCA2-independent manner (Yata et al., 2012). A similar mechanism has been recently found for MRE11 but with a very different outcome. Phosphorylation by Plk1 first led to CK2 phosphorylation of MRE11, rendering it unable to bind DNA and perform repair, which could potentially shut down the repair (Li et al., 2017).

A common feature arising from the role of CK2 in DNA damage response is that usually the regulation of CK2 phosphorylation relies on some other factor, such as another kinase, a phosphatase, or spatial and compartmental separation. In the case of FANCD2/FANCI, the most likely answer is that dephosphorylation is the key, because the phosphomimetic mutants are still localized in the nucleus and the phosphorylation events described by ATR on FANCD2 and FANCI could take place after recruitment to DNA, like monoubiquitination, which these phosphorylation events promote, because ATR has been shown to be recruited to sites of damage (Cuadrado et al., 2006, Jazayeri et al., 2006). However, the exact order of events is not yet fully understood.

Perhaps not surprisingly given the important roles of CK2 in the DNA damage response as well as in other signal transduction pathways, CK2 is being exploited in the treatment of cancer. The CK2 inhibitor CX-4945 is already in clinical trials for the treatment of tumors such as cholangiocarcinoma (Chua et al., 2017), in combination with the crosslinking agent cisplatin. Therefore, a better understanding of the roles of CK2 in ICL repair will be important to improve the treatment of certain cancers. However, as discussed, the activation of the phosphorylated FANCD2/FANCI complex must take place through dephosphorylation, so targeting the phosphatase responsible could also open new therapeutic approaches, maybe in combination with ICL-inducing agents, to inhibit the DNA damage response more efficiently.

### Roles of FANCD2 and the FANCD2/FANCI Complex Outside of ICL Repair

Although we have focused on ICL repair in this study, there is mounting evidence for roles of FANCD2 and the FANCD2/FANCI complex in numerous other processes (Federico et al., 2018, Palovcak et al., 2017). In many of these, DNA binding is essential for the function of FANCD2, so phosphorylation of FANCD2 by CK2 or other kinases could also play important roles regulating DNA binding in these instances.

A link between the FA pathway and DNA replication was one of the first functions to be identified. Components of the FA core complex, as well as FANCD2, are recruited to chromatin during S phase in the absence of induced DNA damage (Mi and Kupfer, 2005, Wang et al., 2008). These FA proteins are needed for replication fork restart after chemically induced collapse (Wang et al., 2008). FANCD2 was shown to facilitate stabilization and restart of replication forks in combination with other repair factors (such as RAD51 and BRCA1/2; Schlacher et al., 2012) and, interestingly, also with nucleases, mechanisms reminiscent of proposed functions of FANCD2 in ICL repair. FANCD2 has been shown to associate with stalled replication forks, in an ATR-dependent manner, through association with the MCM2-MCM7 replicative helicase (Lossaint et al., 2013). FANCD2 then mediates recruitment of CtIP, MRE11, and FAN1 to stalled replication forks and regulates their nucleolytic activity, promoting fork restart but limiting fork degradation (Chaudhury et al., 2014, Lachaud et al., 2016, Yeo et al., 2014). There is also mechanistic evidence for a role of the FANCD2/FANCI complex at stalled forks. It was recently reported that FANCD2/FANCI stabilize RAD51-DNA filaments at stalled forks, and this protects them from degradation by FAN1 (Sato et al., 2016).

Some genomic regions are particularly subject to replication stress, which can lead to breaks and genomic instability. These regions are called common fragile sites (CFSs) and constitute hotspots for genomic deletions and translocations. FANCD2 associates with these regions, and it was recently shown to facilitate replication through CFS by inhibiting dormant origin firing and DNA:RNA hybrid formation that follow replication stalling at CFS (Howlett et al., 2005, Madireddy et al., 2016). The formation of DNA:RNA hybrids or R-loops during transcription is a normal process, but it can cause damage when replication forks encounter them (Okamoto et al., 2019). The FA pathway has been linked also to the resolution of these replication-transcription conflicts. FANCM has been shown to promote R-loop resolution through its translocase activity, and lack of FANCD2 promotes the accumulation of R-loops, pointing to a FANCD2-dependent R-loop resolution also in place (García-Rubio et al., 2015, Schwab et al., 2015).

When replication stalling is not resolved, it can lead to under-replicated regions linking two sister chromatids during mitosis. These linkages form ultra-fine bridges (UFBs) of DNA that can be detected through the presence of proteins such as BLM, RPA, and PICH (PLK1-interacting checkpoint helicase). Interestingly, FANCD2 forms foci at the bases of this type of UFB, but the role of FANCD2 in these structures is still unknown (Chan et al., 2009, Chan et al., 2018).

Some FA proteins, including FANCD2, have also been shown to play a role in the alternative lengthening of telomeres (ALT), a telomere maintenance process independent of telomerase that relies on HR present in some tumor cells. FANCD2 colocalizes with ALT telomeres in a FANCL- and ATR-dependent manner (Fan et al., 2009, Spardy et al., 2008). The exact role of FANCD2 in ALT is still unclear, but it was recently proposed to contribute to resolve replication forks stalled at ALT telomeres and regulate the loading of BLM, which leads to the resection and chromosome exchanges in ALT (Root et al., 2016).

Because DNA binding is vital in all of these processes, it will be interesting to understand whether FANCD2 phosphorylated by CK2 or other kinases can still perform these functions or a similar activation by dephosphorylation by process-specific phosphatases is needed. This could help determine whether the described phosphorylation switch is a master regulator of the activity of FANCD2 or whether it is specific for its role in ICL repair.

### Conclusions

We have described a novel mechanism controlling the DNA loading of the FANCD2/FANCI complex, in turn switching the FA pathway on and off (Figure 6). However, several questions remain. For instance, what is the exact phosphorylation status of the FANCD2/FANCI complex in the cell before and after DNA damage, and how large a fraction of the cellular complex is phosphorylated? A quantitative approach using MS/MS has proved difficult for a six-residue cluster, because multiple combinations of phosphorylated peptides can co-exist at any one point, making their detection especially complicated. Probably only a fraction of the free FANCD2/FANCI complex is dephosphorylated to allow its loading onto DNA and repair to initiate. Also, the precise mechanism behind the changes in DNA binding is also open to further study. The phosphorylated cluster could directly repel the DNA through electrostatic force. Positively charged regions have been identified on the FANCD2 C-terminal Tower domain, which contribute to DNA binding (Liang et al., 2016). Thus, an alternative mechanism could be envisioned whereby the phosphorylated cluster resides in a mobile loop able to bind to this region or another similarly positively charged region (e.g., residues 857–876; Niraj et al., 2017) thereby blocking the DNA binding via occlusion, effectively operating like a DNA-mimetic loop. It is also possible that a larger conformational change takes place whereby, for instance, the Tower domain or another domain rearranges and closes the complex, thus creating an open versus closed conformation. Future experimentation is needed to uncover the actual mechanism.

## STAR★Methods

### Key Resources Table

**Table:** 

REAGENT or RESOURCE	SOURCE	IDENTIFIER
**Antibodies**		

Mouse monoclonal anti-FANCD2 (FI17)	Santa Cruz Biotechnology	Cat# sc-20022; RRID:AB_2278211
Rabbit polyclonal anti-FANCI (G4270)	Merck-Millipore	Cat# ABE1817
Mouse monoclonal anti-α-Tubulin (DM1A)	Merck-Millipore	Cat# 05-829; RRID:AB_310035
Goat polyclonal anti-Lamin B (C20)	Santa Cruz Biotechnology	Cat# Sc-6216; RRID:AB_648156
Mouse monoclonal anti-UHRF1 (H8)	Santa Cruz Biotechnology	Cat# Sc-373750; RRID:AB_10947236
Rabbit monoclonal anti-cyclin A2 (E399)	Abcam	Cat# ab32498, RRID:AB_731777
Mouse monoclonal anti-FLAG (M5)	Sigma-Aldrich	Cat# F4042; RRID:AB_439686
Mouse monoclonal anti-HA (12CA5)	Nakatani and Ogryzko, 2003	N/A
Anti-IL-2 Rα Antibody, clone 7G7/B6	Merck-Millipore	Cat# 05-170; RRID:AB_309642
Rabbit Anti-Mouse Immunoglobulins/HRP	Dako-Agilent	Cat# P0260; RRID:AB_2636929
Goat Anti-Rabbit Immunoglobulins/HRP	Dako-Agilent	Cat# P0448; RRID:AB_2617138
Mouse anti-goat IgG-HRP	Santa Cruz Biotechnology	Cat# sc-2354; RRID:AB_628490

**Bacterial and Virus Strains**		

Top10	Thermo Fisher	Cat# 404010
DH5α	Thermo Fisher	Cat# 18265017
BL21	Thermo Fisher	Cat# 6020-03
DH10Bac	Thermo Fisher	Cat# 10361012

**Chemicals, Peptides, and Recombinant Proteins**		

FuGENE6	Promega	Cat# E2691
Cellfectin II	Thermo Fisher	Cat# 10362100
Protein A Sepharose® Cl^−^4B	Thermo Fisher	Cat# GE17-0963-02
Dynabeads Goat Anti-Mouse IgG	Thermo Fisher	Cat# 11033
Anti-FLAG M2 agarose resin	Sigma-Aldrich	Cat# A2220
Ni^2^ –NTA agarose resin	QIAGEN	Cat# 30210
Trioxsalen (TMP)	Sigma-Aldrich	Cat# 6137
Mitomycin C from *Streptomyces caespitosus*	Sigma-Aldrich	Cat# M4287
CK2 inhibitor (CX-4945)	Biovision	Cat# 2459
Flag-HA-UBA1	Liang et al., 2016	N/A
UBE2T	Liang et al., 2016	N/A
Flag-HA-FANCL	Liang et al., 2016	N/A
His-Ubiquitin	Liang et al., 2016	N/A
Flag-HA-FANCD2 (WT, 6A and 6D forms)	This paper	N/A
His-FANCI	Liang et al., 2016	N/A
Casein kinase II (CK2)	NEB	Cat# P6010S
Benzonase	Sigma-Aldrich	Cat# E1014
Lambda protein phosphatase (Lambda PP)	NEB	Cat# P0753S

**Experimental Models: Cell Lines**		

HeLa FANCD2−/−	Liang et al., 2016	N/A
HeLa Flag-HA-FANCD2 knock-in (partial)	This paper	N/A
HeLa FANCD2−/− +EGFP-FANCD2 (WT, 6A and 6D) + mCherry-UHRF1	This paper	N/A
HeLa FANCD2−/− +Flag-HA-FANCD2 (WT, 6A and 6D)	This paper	N/A

**Oligonucleotides**		

CRISPR primers targeting FANCD2 exon 2 5′CACCCATGC CTCACACATTGCTAC 3′	This paper	N/A
CRISPR primers targeting FANCD2 exon 2 5′AAACGTAGC AATGTGTGAGGCATG 3′	This paper	N/A
ICL14 (+): 5′CATTGTGAATTCGCCTCTCTGTCTAGCCG AAGCTCGAAACGATCTTGTGC-3′	Liang et al., 2016	N/A
ICL14 (-): 5′GTCCATCAAAGTTCGACTGTGCGGCTAGACA GAGAGGCGAATTCACAATG3′	Liang et al., 2016	N/A

**Recombinant DNA**		

pOZ-N	Nakatani and Ogryzko, 2003	N/A
pSpCas9(BB)-2A-Puro (PX459)	Addgene	Cat# 48139
pFastBac1	Thermo-Fisher	Cat# 10359-016
pBlueScript II SK (+)	Addgene	Cat# 212205
pOZ-EGFP-N	Liang et al., 2016	N/A
pOZ-Puro-mCherry	Liang et al., 2016	N/A
pFB-Flag-HA	Liang et al., 2016	N/A
pFB-HTC	Liang et al., 2016	N/A

### Contact for Reagent and Resource Sharing

Further information and requests for resources and reagents should be directed to and will be fulfilled by the Lead Contact, Martin A. Cohn (martin.cohn@bioch.ox.ac.uk).

### Experimental Model and Subject Details

#### Cell Lines

HeLa cells (female) were grown in DMEM (D5796, Sigma) supplemented with 10% FBS. EGFP-fused FANCD2 and mCherry-fused UHRF1 cDNA were expressed using a derivative of the pOZ-N plasmid and sorted using α-IL2R coupled magnetic beads (Dynabeads goat anti-mouse IgG) as described in (Nakatani and Ogryzko, 2003). Transfections of plasmid DNA were carried out using FuGENE6 (Promega) according to the manufacturer’s instructions.

### Method Details

#### Immunoblotting

Antibodies used were as follows: anti-FANCD2, 1:200 dilution (sc-20022, Santa Cruz Biotechnology); anti-α-Tubulin, 1:2000 dilution (5829, Millipore); anti-FANCI, 1:500 dilution (G4270, Merck-Millipore); anti-Lamin B, 1:1000 dilution (sc-6216, Santa Cruz Biotechnology); anti-UHRF1, 1:1000 dilution (sc-373750, Santa Cruz Biotechnology), anti-cyclin A2, 1:2000 (ab32498, Abcam) and anti-Flag, 1:1000 (M5, F4042, Sigma-Aldrich).

#### CRISPR-Cas9 Gene Editing

HeLa FANCD2 −/− cells were generated as described (Liang et al., 2016). HeLa Flag-HA-FANCD2 knock-in cells were generated using plasmid pX459 (Addgene #48139) as described in (Ran et al., 2013). The targeting sequence used in the sgRNA was: 5′-CATGCCTCACACATTGCTAC-3′. Primers: 5′-CACCCATGCCTCACACATTGCTAC-3′ and 5′-AAACGTAGCAATGTGTGAGGCATG-3′ were annealed and introduced into the pX459 plasmid through its BbsI site. A donor plasmid was generated using pBS-SK (+) as the backbone containing 1000bp homology arms upstream (cloned with primers: 5′-CACTTTGGGAGTCCGAGG-3′ and 5′-TTTGACCAATGTCTTGTGC-3′) and downstream (cloned with primers: 5′-ATGGTTTCCAAAAGAAGACTG-3′ and 5′-AATCACCACTAGAGAACTTATTTATG-3′) of exon 2 of *hFANCD2* gene. HeLa cells were co-transfected with 2 μg of pX459 plasmid and 2 μg of donor plasmid and selected after 24 h with 4 μg/ml puromycin. After 24h cells were plated at low densities and clones were picked after 2 weeks. Clones were tested by immunoblot analysis.

#### Cell Cycle Analysis (FACS)

Cells were treated with 20 ng/ml MMC for 2 h and then washed and left to recover for 24 h, or left untreated as a control. Alternatively, cells were subjected to a double thymidine block: 2 mM thymidine was added for 18 h, cells were washed and left in fresh media for 8h and then 2 mM thymidine was added again for 16 h. Cells were then harvested at the indicated time-points and fixed in 70% ethanol at 4°C for 18 h. Cells were then washed 3 times with PBS complemented with 1 mM EDTA at 4°C. Cells were resuspended in PBS containing 1 mM EDTA, 0.1% Triton X-100, 0.2 mg/ml RNase A and 20 μg/ml propidium iodide and incubated at room temperature for 30 min. Samples were processed with a FACSCalibur flow cytometer and data acquired and analyzed with CellQuest software.

#### Immunoprecipitation of Flag-HA-FANCD2 from Knock-in HeLa Cells

Cells were harvested and resuspended in equal pellet volume of buffer 1 (1% Triton X-100, 20 mM Tris (pH 7.5), 5 mM MgCl_2_, 10% glycerol, 10 mM NaF and 2 mM Na_3_VO_4_, freshly complemented with 0.2 mM PMSF, 10 mM NEM and 1U/μl benzonase). Samples were incubated on ice for 10 min with occasional vortex. 10 times pellet volume of buffer 2 (1% Triton X-100, 20 mM Tris (pH 7.5), 10% glycerol, 700 mM KCl, 10 mM NaF, 2 mM Na_3_VO_4_ and 5 mM EDTA, freshly complemented with 0.2mM PMSF and 2mM β-mercaptoethanol) was added. Samples were incubated on ice for 10 min with occasional vortex. The mixture was centrifuged at 13,300 rpm for 10 min at 4°C. Supernatant was collected and incubated with M2 beads (25% pellet volume) at 4°C for 2 h. The mixture was then centrifuged at 2,000 rpm for 5 min at 4°C to pellet down the beads. Beads were recovered and washed once with buffer 2 and twice with buffer 3 (0.1% Triton X-100, 20 mM Tris (pH 7.5), 10% glycerol, 100 mM NaCl, 10 mM NaF, 2 mM Na_3_VO_4_ and 5 mM EDTA, freshly complemented with 0.2mM PMSF and 2mM β-mercaptoethanol). Beads were then incubated for 2 h at 4°C with elution buffer (buffer 3 complemented with 0.5 mg/ml Flag peptide). Elution was then recovered and incubated with α-HA (12CA5) coupled beads (protein A Sepharose) for 2 h at 4°C. Mixture was centrifuged at 2,000 rpm for 5 min at 4°C to pellet down the beads and washed twice with buffer 3. Final elution was performed with 1% SDS and 20 mM DTT at 37°C for 10 min. The elution was concentrated by TCA precipitation: equal volume of 20% TCA was added, mixed, and incubated 18 h at 4°C. Mixture was centrifuged at 13,000 rpm for 30 min at 4°C, pellet was washed with ice-cold acetone and dried. Pellet was then resuspended in 1x SDS-loading dye.

#### Preparation of Radiolabeled DNA Substrates

DNA molecules used for EMSA were prepared as described (Liang et al., 2016). In brief, the DNA oligos were annealed in a buffer containing 10mM Tris-HCl (pH 7.5), 100mM NaCl and 1mM EDTA. ICL14 was created by annealing the following DNA oligonucleotides: ICL14 (+): 5′-CATTGTGAATTCGCCTCTCTGTCTAGCCGAAGCTCGAAACGATCTTGTGC-3′ ; ICL14 (-): 5′-GTCCATCAAAGTTCGACTGTGCGGCTAGACAGAGAGGCGAATTCACAATG-3′.

#### Mass Spectrometric Analysis

Initial phosphorylation events on FANCD2 were identified as follows. Flag-HA-FANCD2 purified from HeLa cells was reduced with DTT, cysteine residues were derivatized with iodoacetamide, and the proteins were separated by SDS-PAGE. Proteins from silver stained gel bands were in-gel digested with trypsin (Shevchenko et al., 1996). The generated peptide mixtures were subjected to LC-MS/MS using a hybrid linear ion trap/ FT-ICR mass spectrometer (LTQ FT, Thermo Electron) essentially as described previously (Haas et al., 2006). MS/MS spectra were assigned by searching them with the SEQUEST algorithm (Eng et al., 1994) against the human International Protein Index sequence database. Subsequent phosphorylation and ubiquitination analysis was performed by the South Parks Road Advanced Proteomics Facility.

#### Protein Purification

UBA1 and FANCL proteins purified from Sf9 cells were expressed using the pFastBac1 vector (Life Technologies) with an engineered N-terminal Flag-HA tag. Cell pellets were resuspended in lysis buffer (20 mM Tris-HCl (pH 8.0), 0.1 M KCl, 10% glycerol, 0.1% Tween-20, 2 mM β-mercaptoethanol and 0.2 mM PMSF). Lysates were clarified by centrifugation, and the supernatants were incubated with M2 anti-Flag agarose resin for 2 h. The resin was washed extensively, and the protein was eluted in the same buffer containing 0.5 mg/ml Flag peptide, however excluding Tween-20. For FANCD2/FANCI complex, Sf9 cell pellets were re-suspended in lysis buffer (20 mM Tris-HCl (pH 8.0), 0.1 M KCl, 10% glycerol and 0.2 mM PMSF), and sonicated. Lysates were clarified by centrifugation (17,000 g), and the supernatants were incubated with M2 anti-FLAG agarose resin (A2220, Sigma) for 2 h. The resin was washed extensively, and the protein was eluted in the same buffer containing 0.5 mg/ml Flag peptide. UBE2T was expressed in *E. coli* BL21 cells. Cells were cultured in Lysogeny broth (LB) supplemented with antibiotics. Once OD_600_ had reached 0.6, protein expression was induced by the addition of 0.5 mM isopropyl-1-thio-b-d-galactopyranoside (IPTG). Cells were cultured overnight at 16°C and harvested the following day by centrifugation. Harvested cells were lysed by sonication of 4 X 10 s bursts on ice, in buffer containing 0.5 M NaCl, 0.1 M Tris (pH 8), 0.02 M imidazole and 0.25 mM tris(carboxyethyl)phosphine (TCEP). Cell debris was removed by centrifugation at 32,000 g. Supernatants were added to equilibrated Ni-NTA agarose (QIAGEN) and incubated on a roller for 1 h at 4°C. 6xHis-Smt3 tags were removed overnight at 4°C by Ulp1 protease at a w/w ratio of 1:15, Ulp1: protein. His-Ubiquitin was purified as described (Liang et al., 2016).

#### *In Vitro* Protein Binding Assay

Recombinant proteins were expressed and purified from Sf9 insect cells as indicated above. 4 μg of Flag-HA-FANCD2/His-FANCI and 1 μg Flag-HA-FANCL were mixed in the reaction buffer containing 100 μg/ml BSA (NEB), 20mM Tris-HCl (pH 7.5), 100mM KCl, 5% glycerol, 2mM β-mercaptoethanol and 0.2mM PMSF. The mixture was first incubated at 30°C for 1h for protein complex formation. Ni^2^-NTA (30310, QIAGEN) beads were added subsequently, and the mixture was incubated at 4°C with gentle mixing for 30 minutes. The mixture was then transferred to Micro Bio-Spin Chromatography Columns (Bio-Rad), and washed with the reaction buffer supplemented with 0.1% Tween-20. The proteins were eluted in SDS-DTT buffer at 37°C and run on an SDS-PAGE gel.

#### Electrophoretic Mobility Shift Assay (EMSA)

EMSA binding reactions contained indicated amounts of FANCD2/FANCI and 1 nM radiolabeled DNA in 10 μL containing 25mM Tris-HCl (pH 7.5), 100mM NaCl, 1mM EDTA, 6% glycerol, 1mM dithiothreitol (DTT). Binding reaction was left at room temperature for 1h, and 0.025% BPB (bromophenol blue) was added. A 4% polyacrylamide (30:1) 0.4xTBE gel was pre-run at 150 mV for 1h at 4°C. The samples were then loaded into the gel and run at 10 mA (100-200 mV) for 3-4h at 4°C. The gel was then dried and exposed to a photo-stimulable phosphor imager plate.

#### *In vitro* Phosphorylation and Dephosphorylation Assays

Phosphorylation reaction containing FANCD2/FANCI and CK2 (P6010, NEB) were incubated at 30°C for 30 min in 50 mM Tris (pH 7.5), 10 mM MgCl_2_, 2 mM ATP and 2 mM DTT. Dephosphorylation reaction containing FANCD2/FANCI and λPP (P0753, NEB) were incubated at 30°C for 30 min in 50 mM HEPES (pH 7.5), 100 mM NaCl, 1 mM MnCl_2_ and 2 mM DTT.

#### *In vitro* Ubiquitination Assays

Reaction contained 17 nM UBA1, 0.64 mM UBE2T, 0.372 mM Flag-HA-FANCL, 4.2 mM His-Ub, 0.25 mM FANCD2/FANCI complex or derivatives thereof, 4 mM pBlueScript SKII (+) when indicated, in the following reaction buffer: 50 mM Tris (pH 7.5), 100 mM KCl_2_, 2 mM MgCl_2_, 0.5 mM DTT and 2 mM ATP. Reactions were incubated at room temperature for the indicated time. 6x SDS loading buffer containing DTT was used to terminate reactions. Samples were loaded onto an SDS-PAGE gel and subjected to Coomassie blue staining or immunoblotting.

#### Clonogenic Survival Assay

Cells (200–1,000) were plated in 6-well plates and, after 24h, treated with different dosages of MMC (mitomycin C). Colony formation was scored after 10-14 days using 1% (w/v) crystal violet in methanol.

#### ICL Induction with TMP

Cells were incubated with 2 μg/ml 4,5’,8-trimethylpsoralen (TMP) for 30 min at 37°C and then irradiated with 50 mJ/cm^2^ UVA (365 nm) in the Spectrolinker XL-1500.

#### Preparation of Whole Cell Lysate and Fractionation

Cells were scraped off the dishes, and centrifuged at 1,000 rpm for 5 minutes. Cell pellets were resuspended and incubated in equal volume of Benzonase buffer (2 mM MgCl_2_, 20 mM Tris (pH 8.0), 10% glycerol, 1% Triton X-100 and 12.5 units/ml benzonase (E1014, Sigma)) on ice for 10 minutes. The cells were then lysed by the addition of an equal volume of 2% SDS to reach a final concentration of 1%. Samples were heated at 70°C for 2 minutes. The protein concentration was determined by Bradford assay (Bio-Rad Life Science). For cell fractionation, cell pellets were permeabilized with CSK buffer containing 200 mM NaCl, 10 mM PIPES, 300 mM Sucrose, 1 mM MgCl_2_, 1 mM EDTA and 0.5% Triton X-100 on ice for 10 min. CSK fraction (supernatant) and nuclear pellet were separated by centrifugation at 900 g at 4°C for 10 min. Nuclear pellet was processed the same way as whole-cell lysate described above.

#### Live-Cell Imaging

EGFP-fused FANCD2 and mCherry-fused UHRF1 cDNA were inserted into the pOZ vector as described above. Live cell imaging were carried out with an OLYMPUS IX81 microscope connected to PerkinElmer UltraView Vox spinning disk system equipped with a Plan-Apochromat 60x/1.4 oil objective using Volocity software 6.3 for image capturing. EGFP and mCherry were excited with 488 nm and 561 nm laser lines, respectively. Throughout the experiment, these cells were maintained at 5% CO_2_, and 37°C using a live cell environmental chamber (Tokai hit). Confocal image series were typically recorded with a frame size of 512x512 pixels and a pixel size of 139 nm. For localized DNA damage induction, cells were seeded in glass bottom dish (MatTek) and sensitized by incubation in DMEM supplemented with 10% FBS and 20 μg/ml 4,5′,8-trimethylpsoralen (TMP) for 30 min at 37°C. Microirradiation was performed using the FRAP preview mode of the Volocity software by scanning (each irradiation time was 100 ms) a preselected area (50x3 pixels) within the nucleus 20-75 times with a 405nm laser set to 100% laser power. The mCherry and EGFP intensities at microirradiated sites were quantified using ImageJ with Fiji, and normalized by their intensities before microirradiation.

### Quantification and Statistical Analysis

Statistical parameters, including statistical tests used, number of events quantified, standard error of the mean, and statistical significance, are reported in the figures and in the figure legends. Statistical analysis has been performed using Microsoft Office Excel software, and statistical significance is determined by the value of p < 0.05.

## Supplemental Information

Document S1. Figures S1–S5

Document S2. Article plus Supplemental Information
