# Supplementary material for: Phosphorylation of FANCD2 Inhibits the FANCD2/FANCI Complex and Suppresses the Fanconi Anemia Pathway in the Absence of DNA Damage
Source: Cell Rep. 2019 Jun 4;27(10):2990–3005.e5. doi: 10.1016/j.celrep.2019.05.003 (PMC6581795; doi:10.1016/j.celrep.2019.05.003)
Supplement: Document S1. Figures S1–S5 [file mmc1.pdf]

Cell Reports, Volume *27*

Supplemental Information

Phosphorylation of FANCD2 Inhibits the FANCD2/FANCI Complex and Suppresses the Fanconi Anemia Pathway in the Absence of DNA Damage

David Lopez-Martinez, Marian Kupculak, Di Yang, Yasunaga Yoshikawa, Chih-Chao Liang, Ronghu Wu, Steven P. Gygi, and Martin A. Cohn

Figure S1

A

Flag-HA

FANCD2 gene (open reading frame in exon 2 in green)

...GTCAAAATGGTTTCCAAAAGAAGACTGTCAAA
ATCTGAGGATAAAGAGAGCCTGACAGAAGATG
CCTCCAGTAAGTATCTAGTCATTTGTTGCTTTA
TTTCCTGTAGCAATGTGTGAGGCATGTGA...

PAM Target

FANCD2 gene (Flag-HA in orange, open reading frame in exon 2 in green)

...GTCAAAATGGACTACAAGGACGACGATGACAA
GCTCGATGGAGGATACCCCTACGACGTGCCCGA
CTACGCCGGAGGAATGGTTTCCAAAAGAAGACT
GTCAAAATCTGAGGATAAAGAGAGCCTGACAGAA
GATGCCTCCAGTAAGTATCTAGTCATTTGTTGCTT
TATTTCCTGTAGCAATGTGTGAGGCATGTGA...

PAM Target

B

Sites	Frequency
8	1
10	1
61	2
64	2
126	1
319	2
592	2
594	1
880	1
881	2
882	1
884	2
886	2
891	2
1214	1
1253	1
1257	2
1401	1
1404	2
1407	2
1408	2
1412	2
1435	1

C

S882

T884/S886

S891

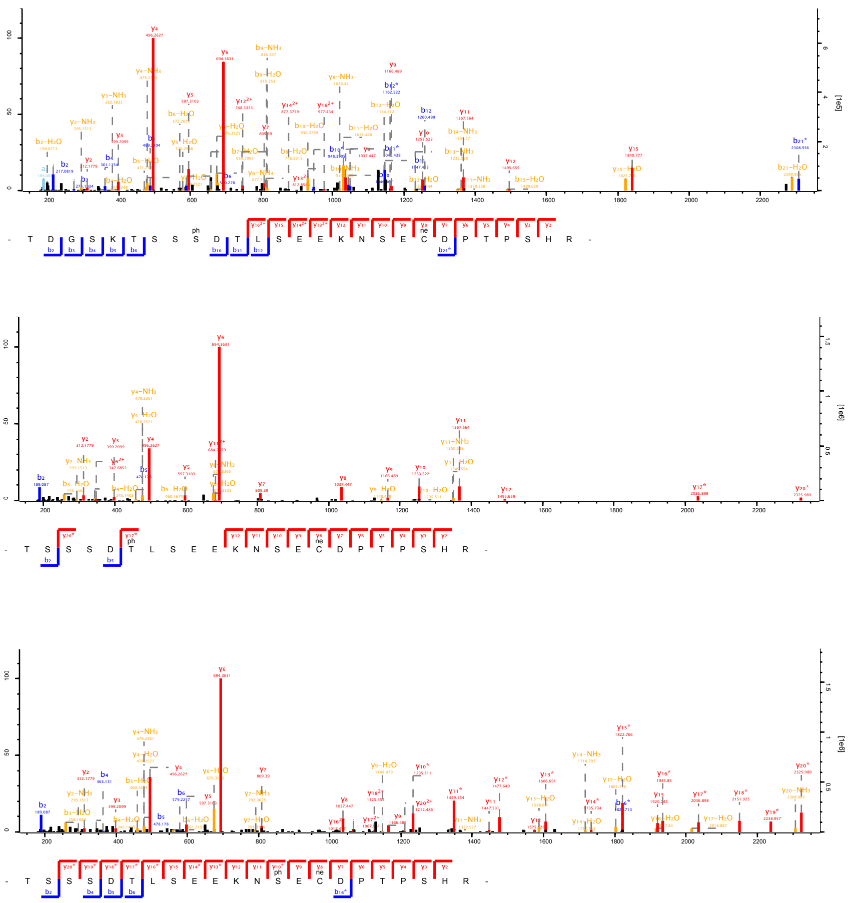

Figure S1 (data relating to Figure 1). (A) Sequence around the open reading frame of exon 2 (green) of the *FANCD2* gene showing the CRISPR target sequence in red and the PAM sequence in blue. The knock-in Flag-HA tag sequence is shown in orange. (B) Table showing the identified phosphosites on FANCD2 in 2 independent MS/MS experiments. (C) MS/MS spectra for the identified phospho-peptides containing the 882-898 cluster on FANCD2.

Figure S2

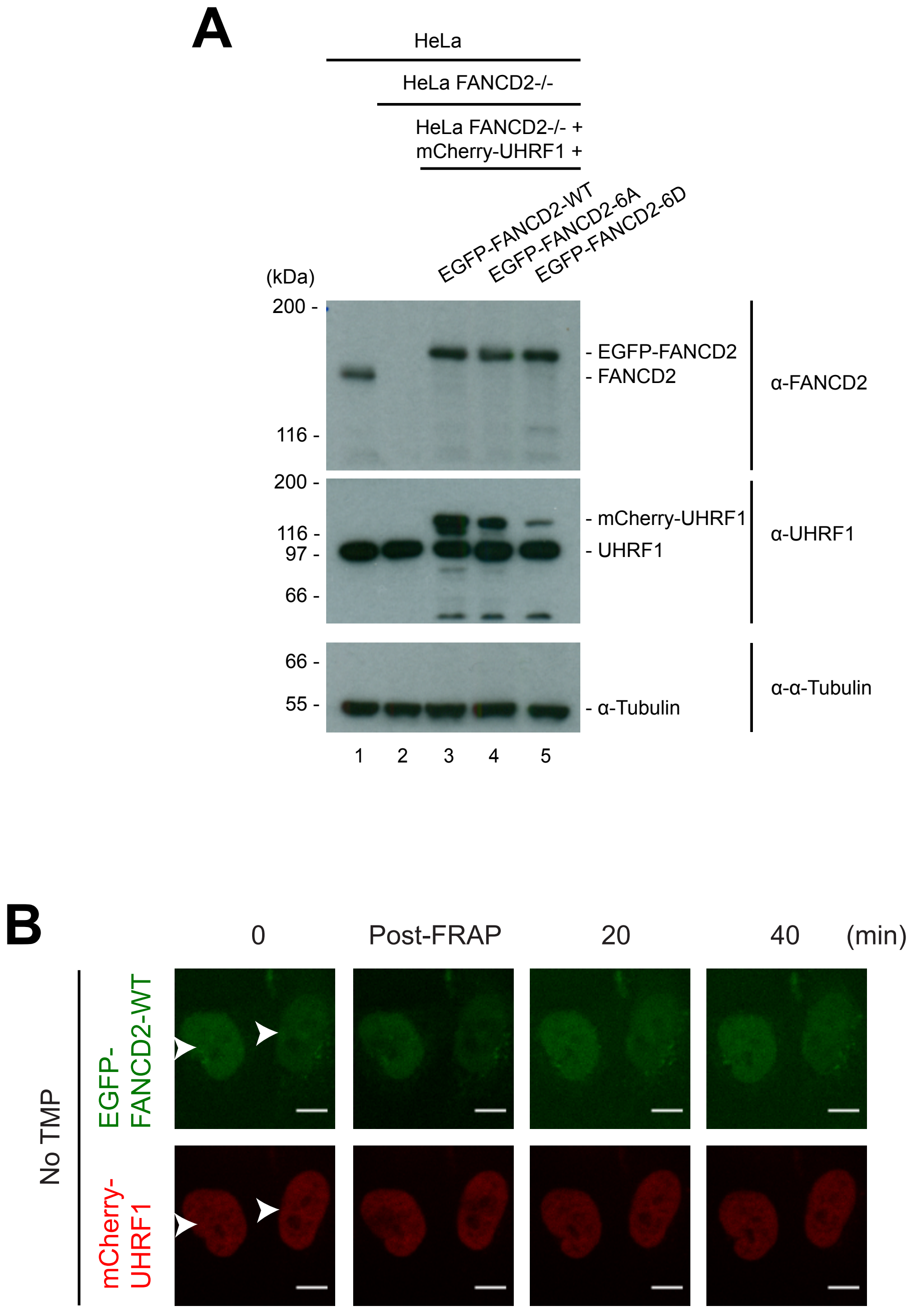

Figure S2 (data relating to Figure 2). (A) Immunoblot analysis showing the expression levels of FANCD2 and UHRF1 in the cell lines used. (B) Live-cell imaging of HeLa FANCD2 -/- cells complemented with EGFP-FANCD2 and mCherry-UHRF1. Cells were microirradiated at the indicated areas (white arrows) in the absence of TMP and followed for the indicated times (scale bar = 10 µm).

Figure S3

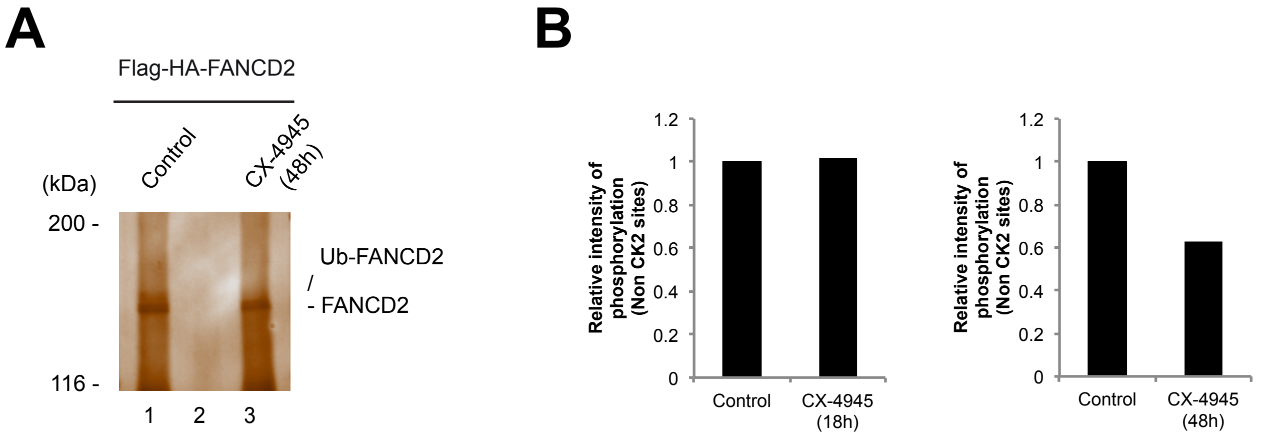

Figure S3 (data relating to Figure 3). (A) Flag purification of endogenous FANCD2 from HeLa S3 knock-in cell line. Cells were either untreated or treated with 10 μM CK2 inhibitor CX-4945 for 48 h. (B) Relative intensity of phosphorylated peptides not containing CK2 sites on FANCD2 in either untreated control or treated with CK2 inhibitor, CX-4945, for 18 h or for 48 h.

Figure S4

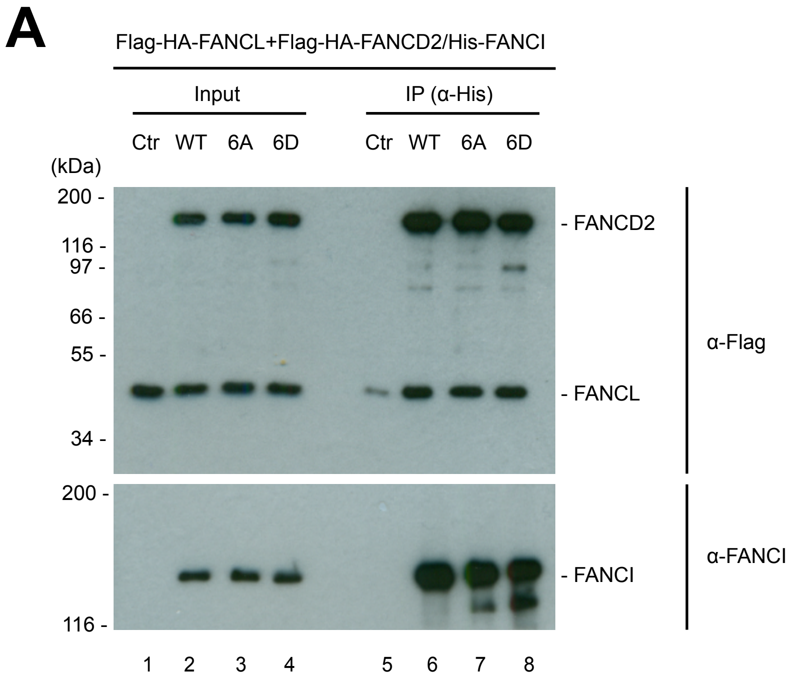

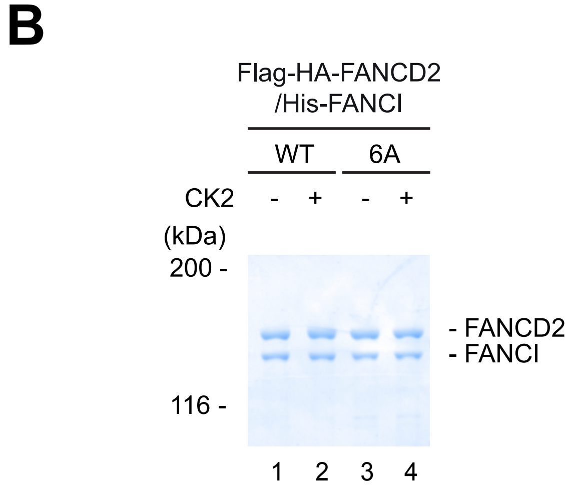

Figure S4 (data relating to Figure 4). (A) Immunoblot analysis showing the in vitro IP of the Flag-HA-FANCD2/His-FANCI complex (WT, 6A or 6D forms) and Flag-HA-FANCL. (B) Coomassie gel of the proteins used in figure 4B, FH-FANCD2-WT/His-FANCI and FH-FANCD2-6A/His-FANCI in mock and CK2 treatments.

Figure S5

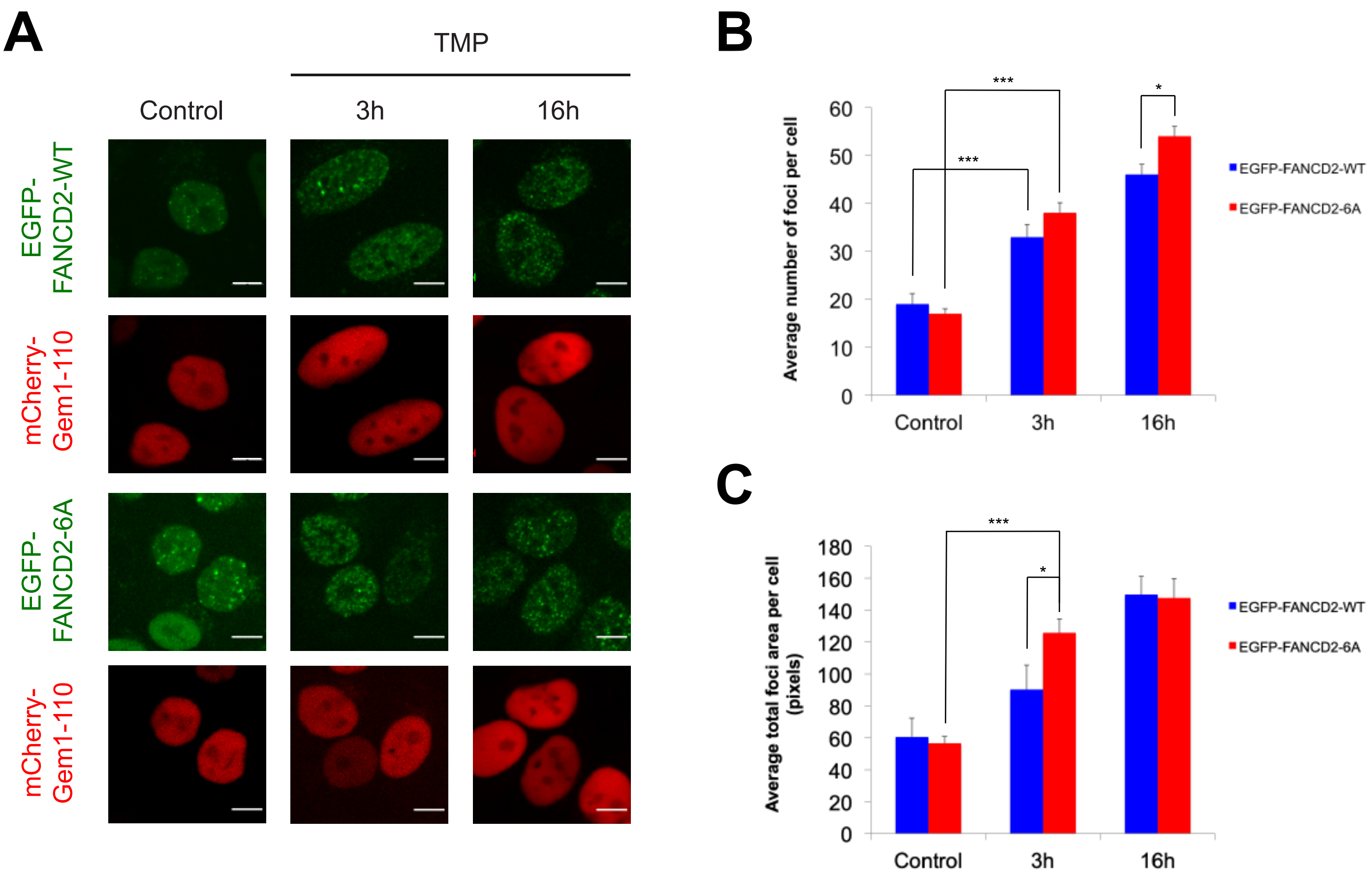

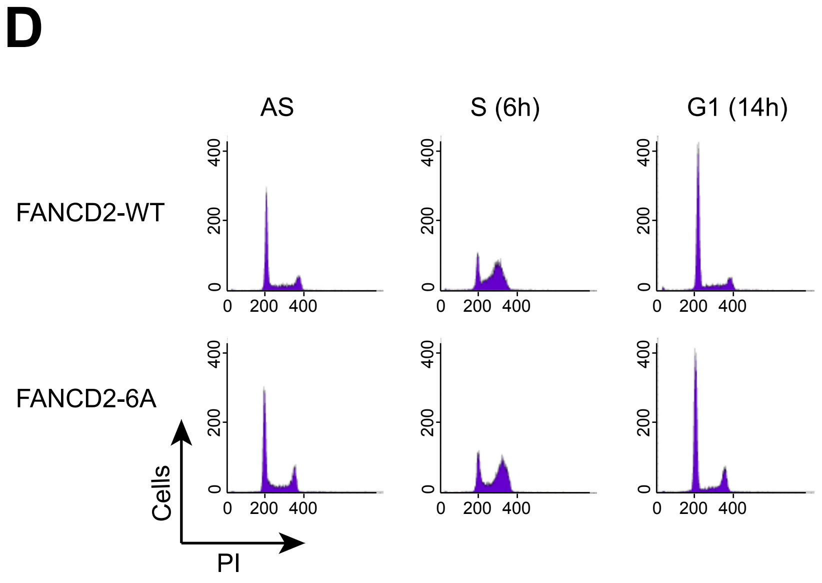

	G1 (%)	S (%)	G2 (%)
**WT AS**	62.3	18.6	19.2
**WT S**	23.7	75.6	0.7
**WT G1**	75.8	13.9	10.3
**6A AS**	60.5	17.6	21.9
**6A S**	27.4	72.3	0.2
**6A G1**	71.1	9.6	19.3

Figure S5 (data relating to Figure 5). (A) Live-cell imaging of HeLa FANCD2 -/- cells complemented with EGFP-FANCD2 or EGFP-FANCD2-6A and mCherry-UHRF1. Cells were left untreated as control or treated with TMP (200 ng/ml) and irradiated with 50 mJ/cm2 UVA and followed for the indicated times (scale bar = 10µm). (B) Quantification of the average number of foci per cell in each time-point (Mean ± SEM, n = 25). * $p < 0.05$, *** p , 0.001. (C) Quantification of the average total foci area per cell in each time-point (Mean ± SEM, n = 25). * $p < 0.05$, *** p , 0.001. (D) Cell cycle profiles measured by DNA content of HeLa FANCD2 -/- cells complemented with Flag-HA-FANCD2-WT or Flag-HA-FANCD2-6A synchronized with double thymidine block and harvested at the indicated timepoints (AS: asynchronous, S: 6 h after release from thymidine block, G1: 14 h after release from thymidine block).
